# Differentiating between bacterial and viral infections by estimated CRP velocity

**DOI:** 10.1371/journal.pone.0277401

**Published:** 2022-12-07

**Authors:** Michal Largman-Chalamish, Asaf Wasserman, Adi Silberman, Tal Levinson, Omri Ritter, Shlomo Berliner, David Zeltser, Itzhak Shapira, Ori Rogowski, Shani Shenhar-Tsarfaty

**Affiliations:** 1 Department of Internal Medicine C", D" and E", Tel Aviv Sourasky Medical Center, Tel Aviv, Israel; 2 Department of Rheumatology, Tel Aviv Sourasky Medical Center, Tel Aviv, Israel; 3 Infectious Diseases Unit, Tel Aviv Sourasky Medical Center, Tel Aviv, Israel; 4 Department of Emergency Medicine, Tel Aviv Sourasky Medical Center, Tel Aviv, Israel; 5 Sackler Faculty of Medicine, Tel Aviv University, Tel Aviv, Israel; Kaohsuing Medical University Hospital, TAIWAN

## Abstract

**Purpose:**

Differentiating between acute viral and bacterial infection is challenging due to the similarity in symptom presentation. Blood tests can assist in the diagnosis, but they reflect the immediate status and fail to consider the dynamics of an inflammatory response with time since symptom onset. We applied estimated C-reactive protein (CRP) velocity (eCRPv), as derived from the admission CRP level divided by time from symptom onset, in order to better distinguish between viral and bacterial infections.

**Methods:**

This cross-sectional study included patients admitted to the emergency department with a confirmed viral (n = 83) or bacterial (n = 181) infection. eCRPv was defined as the ratio between the absolute CRP level upon admission to time from symptom onset (in hours). Absolute CRP and eCRPv values were compared between the 3 groups.

**Results:**

Bacterial patients presented with higher CRP levels (133 mg/L) upon admission compared to viral patients (23.31 mg/L) (*P* < 0.001). Their median value of eCRPv velocity was 4 times higher compared to the viral patients (1.1 mg/L/h compared 0.25 mg/L/h, *P* < 0.001). Moreover, in intermediate values of CRP (100–150 mg/L) upon admission, in which the differential diagnosis is controversial, high eCRPv is indicative of bacterial infection, eCRPv >4 mg/L/h represents only bacterial patients.

**Conclusions:**

During an acute febrile illness, the eCRPv value can be used for rapid differentiation between bacterial and viral infection, especially in patients with high CRP values. This capability can potentially expedite the provision of appropriate therapeutic management. Further research and validation may open new applications of the kinetics of inflammation for rapid diagnosis of an infectious vs. a viral source of fever.

## Introduction

Bacterial and viral infections cause similar symptoms, such as weakness, fever, muscle pain, and others. Early distinction between those 2 types of infections is essential for appropriate treatment and prognosis. While bacterial infections are usually treated with antibiotics, treating viral infections with antibiotics is ineffective and might promote antibiotic resistance [1]. Both viral and bacterial infections lead to an acute inflammatory response characterized by increasing pro-inflammatory cytokines or chemokines that can spill over into the circulation and result in systemic cytokine storms, which, in turn, can lead to multiorgan dysfunction [2, 3]. C-reactive protein (CRP) is an acute-phase protein that serves as an early marker of inflammation or infection. The protein is synthesized in the liver and is normally found at concentrations of less than 10 mg/L in the blood. During infectious or inflammatory disease states, CRP levels rise rapidly within the first hours and peak at levels of up to 350–400 mg/L after 48 hours [4]. CRP is a real-time and low-cost biomarker that serves as a screening tool in the emergency department (ED), and a high CRP concentration is indicative of a bacterial infection [5]. This phenomenon is described by the binding capability of CRP to phosphorylcholine, which exists only with bacteria [6–8]. Moreover, high CRP levels are associated with hospital re-admissions and infection severity [9, 10].

A relatively new approach is to study the level of inflammatory biomarkers in relation to disease duration for exploring the kinetics of inflammation. A recently published study by our lab showed the ability of those kinetics between 2 consecutive CRP measurements to distinguish between viral and bacterial infections [11]. We demonstrated its usefulness for patients with low levels of CRP upon admission, among whom a high rate of the development of an acute response over time is indicative of a bacterial infection [11]. Another study from our lab had shown that the use of estimated CRP velocity (eCRPv), which is the ratio between the absolute CRP concentration upon admission and the time since symptom onset (in hours) instead of the absolute concentration could enhance the ability to distinguish bacterial infections from non-bacterial infections [12]. According to this trend, in our study, we try to achieve a new method that helps physicians to distinguish between viral and bacterial infection. The novelty of our study is that the differentiation can be made in the admission with one measurement and anamnestic detail about the onset of symptoms. The rationale for using the value of eCRPv stems from the assumption that severe infections might be associated with a rapid cytokine storm.

The inflammatory response plays a critical role in the coronavirus disease 2019 (COVID-19) and influences the progression of the disease. Moreover, an inflammatory cytokine storm increases the severity of COVID-19 [13]. CRP is one of the inflammatory markers that can effectively assess disease severity. High levels of CRP are associated with the development of severe disease, and they are correlated with pulmonary lesions at the early stage of COVID-19 [14, 15]. We reasoned that studying the dynamics of CRP by eCRPv would also advance our understanding of the progression of COVID-19.

Our current study addresses the dynamics of CRP by determining eCRPv values with the aim of discriminating between viral and bacterial infections. Our study cohort includes patients who presented to the ED with fever and suspected infection. We measured each patient’s absolute CRP values and recorded the self-reported time since the onset of symptoms. We focused upon groups of patients with a similar range of CRP concentration (iso-CRP groups) and provided the probability of the infection being bacterial in origin in each group.

## Material and methods

### Study population

We conducted a cross-sectional study of patients admitted to the Tel-Aviv Sourasky Medical Center ED with fever and suspected infection between February 2018 to March 2020. The study was approved by the medical center’s Helsinki committee and conformed to the principles outlined in the declaration of Helsinki (TLV-17-0590). Informed consent was obtained from all participants. The inclusion criteria were age over 18 years with an established diagnosis of bacterial or viral infection, and the availability of CRP levels in blood samples taken at admission to the ED as part of routine clinical assessment. Bacterial infections were identified by a positive bacterial blood culture (n = 181). Viral infections were identified by either a positive PCR for a virus or an Immunoglobulin test indicative of acute viral infection (n = 83). Excluded were pregnant woman, patients with active malignancy or any inflammatory disease, and patients on a regimen of immunosuppressive therapy or anti-inflammatory medications.

## Methods

The differential diagnosis was performed by two expert internal medicine physicians and one expert in infectious diseases, based upon a combination of laboratory tests and clinical assessments. The source of infection was classified as being definite bacterial or definite viral based upon the isolated agent causing the infection. CRP measurement is part of the routine clinical care in our medical center. The first CRP measurement was taken during the patient’s admission to the emergency department. The study took place during the patient’s admission before hospitalization. We chose this earliest possible time point because the main objective was to help physicians to accurately distinguish between bacterial and viral infections which is crucial in deciding on antibiotic treatment and institution of sepsis protocols if needed.

The main dependent variable of the study was a viral vs a bacterial infection diagnosis. The independent variables were CRP and eCRPv levels, age, sex, and the comorbidities of hypertension or diabetes mellitus. eCRPv was defined as the ratio between the absolute CRP value at ED admission and time from self-reported symptom onset (in hours).

### Statistical analysis

All continuous variables are displayed as means (±standard deviation (SD)) for normally distributed variables or median (interquartile range (IQR)) for variables with abnormal distribution. Categorical variables are displayed as numbers (%) of subjects within each group. Continuous variables were compared by a student’s t-test for normally distributed variables and by the Kruskal-Wallis and Mann-Whitney tests were used to compare for non-normally distributed ones (i.e CRP levels or eCRPv values between categories (viral vs. bacterial)). To assess associations among categorical variables, we used a Chi-square test. We assessed normal distributions using Kolmogorov–Smirnov’s test and Q-Q plots. All of the statistical tests were 2-tailed, and a *P* value <0.05 was considered statistically significant. All statistical analyses were performed using the IBM SPSS Statistics 25 statistical package (IBM Corporation, Armonk, New York, USA).

## Results

### Patient demographics

Our study included 181 patients with confirmed bacterial infection, and 83 patients with confirmed viral infection. The patients diagnosed as having a bacterial infection were older compared to the patients diagnosed as having a viral infection (57.79 vs 43.38 years of age, respectively), had twice the prevalence of hypertension (43.7% vs 21.7%), and a much higher prevalence of dyslipidemia (37.6% vs 18.1%). The 2 groups were similar in sex, body mass index, and time from symptom onset (Table 1).

**Table 1:** Study population characteristics.

Group	Bacterial	Viral	*P* value
n	181	83	
Age, y (± STD)	57.79 (18.9)	43.4 (19.1)	<0.001
Sex, male	110 (60.8%)	56 (67.5%)	0.225
BMI, kg/m^2^ (± STD)	24.0 (3.1)	23.7 (4.2)	0.500
Time from symptoms, h median (IQR)	96 (48–168)	96 (48–168)	<0.001
Dyslipidemia, %	37.6	18.1	<0.001
Hypertension, %	43.7	21.7	<0.001
CRP admission, median (IQR)	133 (48.99–192.26)	23.31 (8.72–53)	<0. 001
eCRPv, median (IQR)	1.1 (0.4–2.61)	0.25 (0.05–0.78)	<0. 001
WBC, 10^9^/L median (IQR)	11.3 (8.4–14.28)	6.85 (5.07–10.43)	<0. 001
Neutrophil, % median (IQR)	79 (72–87)	68 (50–79)	<0. 001
Lymphocytes, % median (IQR)	13 (8–20)	22 (12–42)	<0.001
PLT, 10^9^/L, median (IQR)	224 (173.5–280)	179 (150–217)	<0.0001

BMI = body mass index, CRP = C-reactive protein, eCRPv = estimated C-reactive protein velocity, WBC = white blood cells, PLT = platelets, IQR = interquartile range, STD = standard deviation.

### Study findings

**Fig 1:**
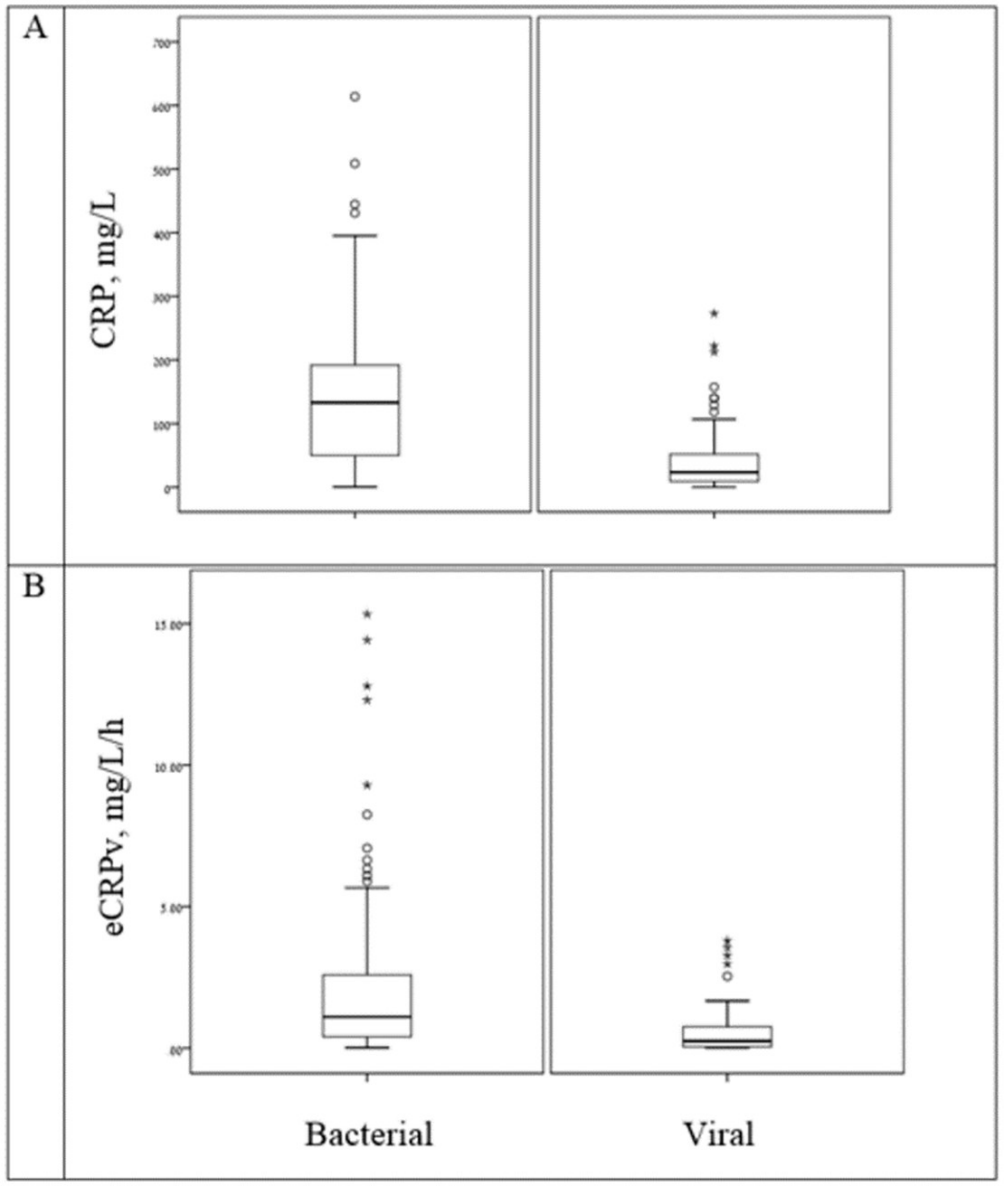
Distribution of CRP (A) and eCRPv (B) according to source of infection. Patients with bacterial infections presented with higher CRP levels upon ED admission and higher eCRPV levels compared to patients with viral infections.

**Fig 2:**
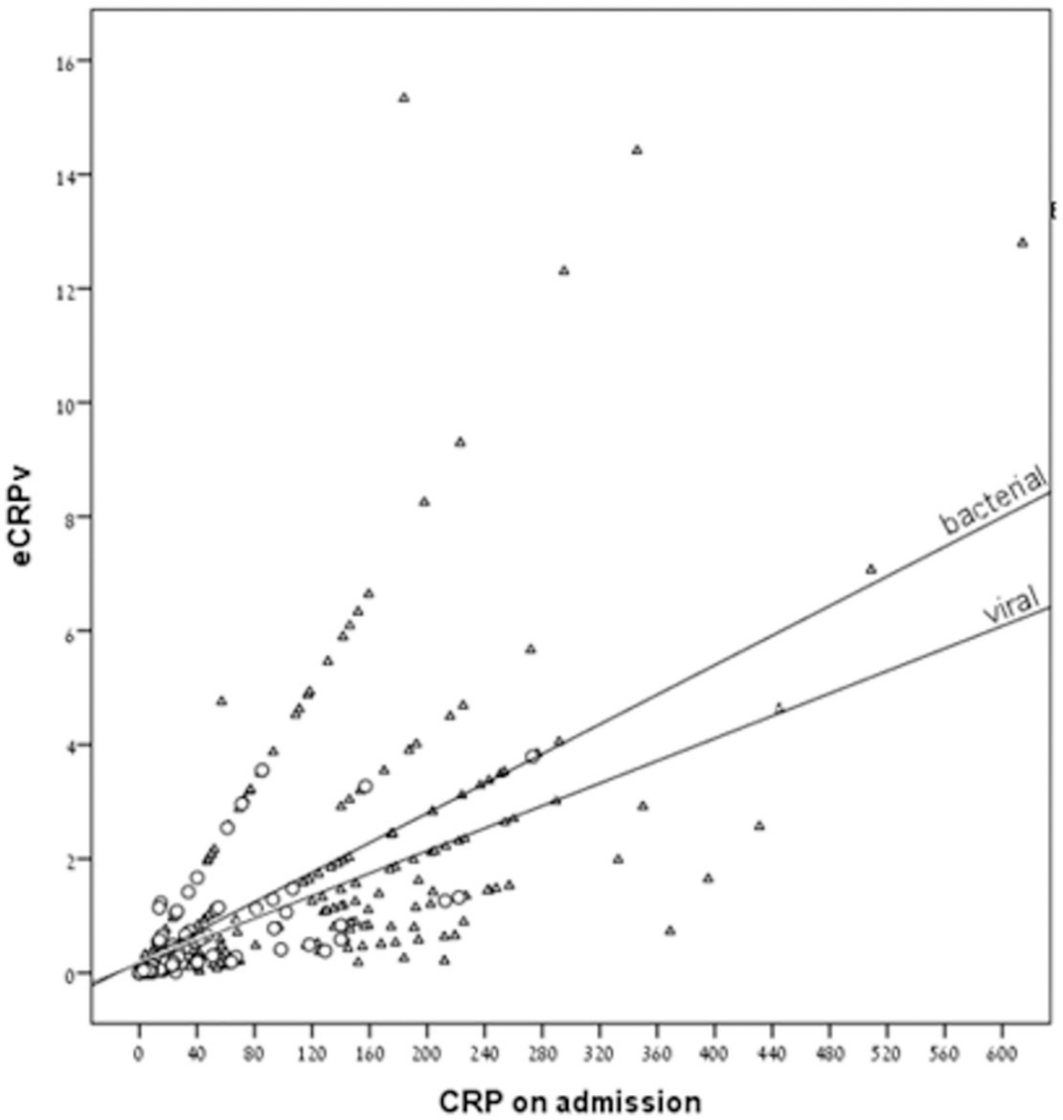
Correlation between absolute CRP concentrations and CRP velocity. Patients with bacterial infections presented with higher correlations between admission CRP (mg/L) and eCRPv (mg/L/h) compared to patients with viral infections. The triangles indicate patients with a bacterial infection and the circles indicate patients with a viral infection.

**Fig 3:**
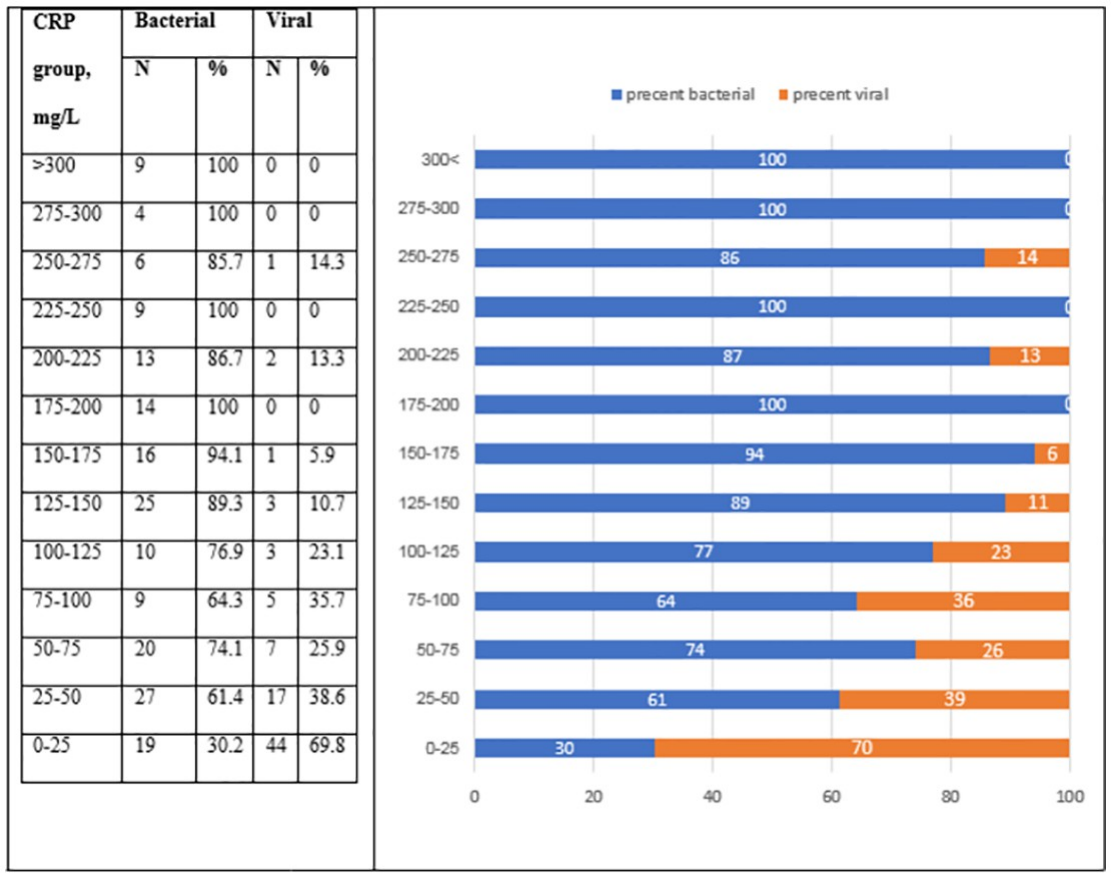
The ratio between bacterial (blue) and viral (orange color) infections in each range of CRP values.

**Fig 4:**
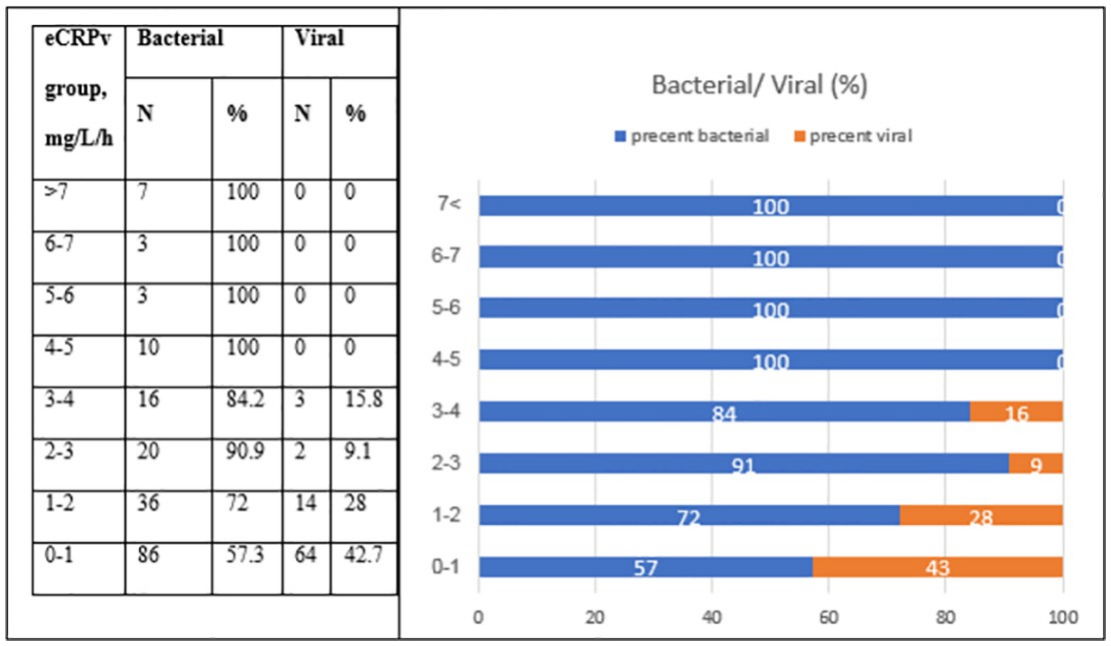
The ratio between bacterial (blue) and viral (orange color) infections in each range of eCRPv values.

As expected, patients with a bacterial infection had higher CRP levels upon ED admission compared to patients with a viral infection (median CRP: 133 and 23.31, respectively, *P* < 0.001) (Table 1 and Fig 1A). The estimated CRP velocity was also 4 times higher in the bacterial group compared to the viral group (median eCRPv: 1.1 and 0.25, respectively, *P* < 0.001) (Fig 1B). The correlation between absolute CRP concentrations and the CRP velocity was higher in the bacterial compared to the viral group (r = 0.29 and r = 0.421, respectively) (Fig 2). The analysis of groups of patients with iso-CRP (i.e., with a similar range of CRP concentration) indicates that in high values of CRP concentration most of the patients have a bacterial infection. Fig 3 presents the ratio between bacterial and viral infections in each range of CRP values. With a single exception, all of the patients who were admitted with CRP levels >275 mg/L were diagnosed as having a bacterial infection. Analysis of the groups of patients with iso-eCRPv values revealed that the source of infection of patients with eCRPv values >4 was bacterial (Fig 4).

**Fig 5:**
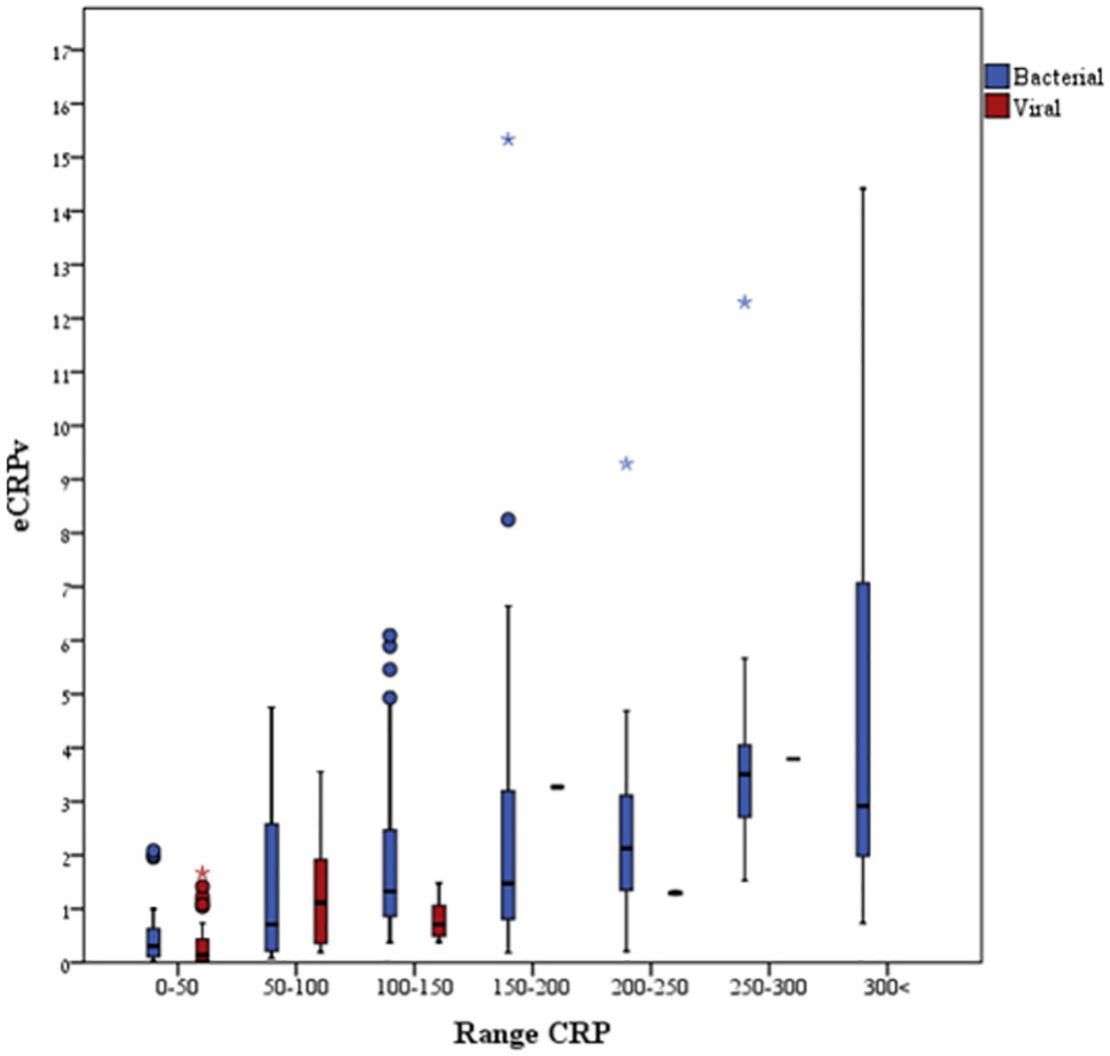
eCRPv values for the groups of patients with iso-CRP levels. Higher eCRPv values were observed almost exclusively among patients with CRP levels >150 mg/L who had validated bacterial infections.

Finally, we compared the median eCRPv values between viral and bacterial infections among the patients in the iso-CRP groups. There was a significant difference between the eCRPv findings of patients with a bacterial infection and those with a viral infection for those with CRP findings in the range of 100–150 mg/L (Table 2 and Fig 5). A high eCRPv was indicative of a bacterial infection for those patients.

**Table 2:** eCRPv per CRP levels (iso-CRP).

CRP groupmg/L	eCRPV, mg/L/h						*P* value
	Total		Bacterial		Viral		
	Median	IQR	Median	IQR	Median	IQR	
>300	2.92	1.81–9.93	2.92	1.81–9.93	-	-	-
250–300	3.52	2.71–4.05	3.50	2.69–4.46	-	-	0.909
200–250	1.79	1.28–3.04	2.13	1.31–3.16	-	-	0.261
150–200	1.56	0.81–3.27	1.48	0.81–3.28	-	-	0.516
**100–150**	**1.18**	**0.82–2.00**	**1.32**	**0.84–2.92**	**0.71**	**0.46–1.16**	**0.028**
50–100	0.79	0.29–2.56	0.71	0.20–2.73	1.11	0.33–2.23	0.596
**0–50**	**0.19**	**0.05–0.53**	**0.31**	**0.10–0.63**	**0.14**	**0.04–0.46**	**0.017**

CRP = C-reactive protein, eCRPv = estimated C-reactive protein velocity, IQR = interquartile range.

**Bold** indicates significant difference of eCRPv between bacterial and viral patients.

## Discussion

We explored the possibility of using the kinetics of CRP (eCRPv) as a possible aid to discriminate between a viral and a bacterial infection. The use of inflammatory response dynamics rather than a single blood test that represents the current state is a step forward for differentiating between acute viral and bacterial infections, which have a similar presentation of weakness, fever, muscle pain, and other symptoms among patients admitted to hospitalization due to infection. We demonstrated that the use of the CRP results and time from symptom onset could support physicians in differentiating between viral and bacterial infections by suggesting that patients with eCRPv values >4 have a 100% chance of having a bacterial source of infection. Furthermore, the eCRPv result provides a better differential diagnosis among patients with high levels of CRP upon admission, with lower eCRPv values being indicative for a viral etiology. Moreover, there is a significant difference in the intermediate values of CRP (100–150 mg/L) between the eCRPv of patients with bacterial and viral infections for which the differential diagnosis is controversial, further demonstrating that a high eCRPv is indicative of a bacterial infection. Nevertheless, in the group of patients with values of CRP between 50–100 mg/L, the eCRPV value of bacterial group did not reach significance level compared to viral group. We suggest to focus on this subgroup population on future research.

Applying the dynamics of an inflammatory biomarker for improving differential diagnosis has been suggested before. For example, the difference between two CRP measurements has been used to distinguish between viral and bacterial infections [11]. However, that study was based upon CRP measurements taken at admission and at a later time point, and so the physician cannot benefit from the results without delay.

To the best of our knowledge, there is no consensus regarding the cutoff for a CRP value to indicate the presence of a bacterial infection with high probability, with cutoffs of 10, 20, and 80 mg/L having been suggested [5, 16, 17]. The prevailing assumption is that the host immune response to the coronavirus plays a critical role in clinical manifestation and disease severity, however, our study was conducted before the era of the COVID-19 pandemic [18, 19]. Calculating the eCRPv value at admission to the ED could contribute to the assessment of the immune response and, accordingly, of the disease severity. Specifically, inflammatory markers that reflect the host response can help the physician when clinical parameters measured upon ED admission are insufficient and the microbiological test results are not yet available. Previous studies presented high concentrations of CRP, procalcitonin (PCT) and interleukin-6 (IL-6) in bacterial infections compared to viral infections [20, 21]. In addition, previous studies showed a low level of platelets in viral infections compared to bacterial infections. Platelets are now recognized as an inflammatory cells with a role in the immune responses, including viral infections [22, 23]. Our study, approved the significant decline in platelets among viral infections.

Our study has strengths and limitations. A major contribution is the parameter of eCRPv which is derived from a simple calculation and one which can assist in the distinction between viral and bacterial infection from the blood sample taken as soon as the patient arrives at the hospital. The limitations of our study include the prospective study design in which we included only those patients who were able to sign an informed consent form, establishing a bias toward less difficult cases. Indeed, our study population includes patients with a severe viral infection and only a mild-moderate bacterial infection, and the inclusion of more patients with a severe bacterial infection could have helped to improve the differentiation between the groups. Another limitation is the choice of study participants who comprised a relatively small group from only one hospital, with 2:1 ratio favoring bacterial infection. As such, further validation of our findings from larger and more heterogeneous populations are warranted. In addition, other chronic underlying disease could also elevate CRP, such as cardiovascular disease, type 2 diabetes mellitus, age-related macular degeneration, hemorrhagic stroke, Alzheimer’s disease, Parkinson’s disease [24] this might alter the results of eCRPv and must therefore be examined in a further study, with a larger population. Lastly, the exact timing from the onset of symptoms was estimated based on the medical record of each patient’s admission file. Hospital records were reviewed manually in order to document time from symptoms onset in hours. This introduced two limitations to our study, the first being a bias limitation, by the fact that we could not retrieve the exact timing of symptom onset from the files, but obtained only a rough estimation based on the patient’s self-report. Another possible limitation could have been in the recording of number of hours from patient’s files. However, the recording was done by the same physician to all patients, using a strict protocol, therefore, we believe our recorded parameter of time from symptoms onset represent a fair estimation.

## Conclusion

We demonstrated that the added extrapolation of eCRPv instead of solely using the absolute CRP concentration in ED admission blood samples could improve the distinction between viral and bacterial infection. eCRPv could become a useful diagnostic aid to rapidly identify patients with a bacterial infection and with no additional costs. Further research and validation in a larger group of patients may open new avenues for the use of the kinetics of inflammatory biomarkers for differential diagnosis of the source of infection.

## Supporting information

S1 Data (XLSX)

S1 Fig (DOCX)

S1 Table Whole cohort population characteristics. (DOCX)
