# Supplementary material for: Differentiating between bacterial and viral infections by estimated CRP velocity
Source: PLoS One. 2022 Dec 7;17(12):e0277401. doi: 10.1371/journal.pone.0277401 (PMC9728869; doi:10.1371/journal.pone.0277401)
Supplement: S1 Fig — (DOCX) [file pone.0277401.s002.docx]

**Supplementary Figure 1**

N = 341

Patients with undetermined etiology, (n = 42).

Patients excluded due to active malignancy, inflammatory disease, immunosuppressive therapy (n = 35).

**Final Cohort**

N _bacterial_ = 181

N _viral_ = 83
