# Supplementary material for: Differentiating between bacterial and viral infections by estimated CRP velocity
Source: PLoS One. 2022 Dec 7;17(12):e0277401. doi: 10.1371/journal.pone.0277401 (PMC9728869; doi:10.1371/journal.pone.0277401)
Supplement: S1 Table — (DOCX) [file pone.0277401.s003.docx]

**Supplementary Table 1.** Whole cohort population characteristics.

| **Parameter** | **Distribution** |
| --- | --- |
| n | 386 |
| Age, y, mean (± STD) | 53.11 (20.59) |
| Sex, males | 239 (61.92%) |
| BMI, kg/m^2,^, mean (± STD) | 24.35 (4.99) |
| Type: n |  |
| Bacterial | 181 (46.89%) |
| Viral | 83 (21.50%) |
| Undetermined | 42 (10.88%) |
| Exclusion | 35 (9.07%) |
| CRP at admission, median (IQR) | 51.5 (11.75- 142.25) |
| eCRPv, median (IQR) | 0.53 (0.13- 1.44) |
| WBC, 10^9^/L, mean (± STD) | 10.09 (4.85) |
| Neutrophil, %, mean (± STD) | 70.56 (15.87) |
| Lymphocytes, %, mean (± STD) | 18.51 (13.08) |
| PLT, 10^9^/L, mean (± STD) | 227.28 (92.04) |

BMI = body mass index, CRP = C-reactive protein, eCRPv = estimated C-reactive protein velocity, WBC = white blood cells, PLT = platelets, IQR = interquartile range, STD = standard deviation.
